# Spatial temporal trends and clustering of mental health in the Great Plains and Rocky Mountains

**DOI:** 10.3389/fpubh.2026.1776208

**Published:** 2026-07-28

**Authors:** Phyllis N. Muniu, Folashade B. Agusto

**Affiliations:** 1 Ecology and Evolutionary Biology, University of Kansas, Lawrence, KS, United States; 2 Mathematics and Applied Mathematics, North-West University, Potchefstroom, South Africa

**Keywords:** GTWR, mental health, MGTWR, Moran's I, socioeconomic disparities, spatial autocorrelation, spatial heterogeneity

## Abstract

Mental health is a critical component of overall well-being, profoundly influencing how individuals think, act, and interact in daily life. In the United States, an estimated 57.8 million adults experience a mental illness, representing 22.8% of the adult population. Access to mental health care is particularly challenging in the Great Plains and Rocky Mountains, where high costs and limited treatment centers exacerbate disparities. Understanding the spatial distribution of mental health outcomes in these regions is essential for guiding resource allocation and informing targeted interventions. In this study, we investigate the spatio-temporal dynamics of frequent mental distress (FMD) across counties in the Great Plains and Rocky Mountains from 2019 to 2023. We examine how socioeconomic, demographic, and healthcare-related variables influence mental health outcomes. To account for spatial dependence and spatiotemporal heterogeneity, we use Moran's I, ordinary least squares (OLS), geographically and temporally weighted regression (GTWR) and multiscale geographically and temporally weighted regression (MGTWR) models. Results revealed persistent spatial clustering of FMD, with higher levels concentrated in counties within Kansas, Nebraska, Oklahoma, and Texas and lower levels observed in North Dakota, Wyoming, and Montana. Significant spatial heterogeneity was identified in the associations between FMD and unemployment, poverty, uninsured population, educational attainment, and racial and ethnic composition. Compared with OLS, both GTWR and MGTWR improved model performance and better captured localized variation in FMD associations. Temporal variation was comparatively limited relative to the pronounced spatial heterogeneity observed across counties. These findings suggest that mental health disparities across the Great Plains and Rocky Mountains are associated with geographically uneven socioeconomic and structural conditions. The study highlights the importance of accounting for spatial non-stationarity in population mental health research and demonstrates the value of geographically weighted approaches for identifying localized disparities and informing targeted public health interventions.

## 1 Introduction

Mental health disorders are among the leading contributors to disability and reduced quality of life worldwide (1, 2). In the United States, poor mental health is associated with increased risks of chronic disease, reduced productivity, higher healthcare utilization, and premature mortality (3, 4). Although mental health challenges affect populations across the country, their burden is unevenly distributed geographically, reflecting differences in socioeconomic conditions, healthcare access, demographic composition, and community environments (5, 6). Understanding the spatial distribution of mental health outcomes is therefore essential for developing targeted public health interventions and reducing geographic health inequalities (7, 8).

Mental health outcomes worsened substantially during the COVID-19 period. Ettman et al. (9) reported a substantial increase in depression symptoms among U.S. adults during the COVID-19 pandemic compared with pre-pandemic estimates, indicating a marked deterioration in population mental health during the early stages of the crisis. Similarly, Daly and Robinson (10) found elevated levels of psychological distress throughout the pandemic, with distress peaking during the initial phase before gradually declining over time.

McGinty et al. (12) and Czeisler et al (11) observed significantly higher levels of psychological distress and loneliness among U.S. adults during the early months of COVID-19 relative to pre-pandemic levels. They reported elevated prevalence of anxiety and depression symptoms, increased substance use, and suicidal ideation among adults in the United States. These findings indicate that the pandemic was associated with widespread deterioration in psychological well-being and behavioral health outcomes across the population. Consequently, the period from 2019 to 2023 provides a unique opportunity to examine changes in mental health before, during, and following the most acute phases of the pandemic.

Racial and ethnic disparities in mental health outcomes remain a persistent public health concern in the United States (13–15). Minority populations often experience greater exposure to socioeconomic disadvantage, discrimination, healthcare barriers, and other structural inequities that contribute to poorer mental health outcomes and reduced access to mental health services (15). Indigenous communities, in particular, face disproportionate mental health burdens associated with historical trauma, geographic isolation, underinvestment in healthcare infrastructure, and limited access to culturally responsive care (16, 17). These disparities are shaped by broader social and structural determinants rather than race or ethnicity alone, highlighting the importance of considering demographic composition alongside socioeconomic and healthcare-related factors when examining geographic variation in mental distress. Given the demographic diversity of the Great Plains and Rocky Mountain regions, racial and ethnic composition may contribute to geographic variation in mental distress and therefore warrants consideration in the analyses of mental health.

The availability and efficacy of mental health care varies greatly among states, exacerbating already serious concerns in the United States (18). Prior county-level research has shown that access to mental health care varies substantially across the United States because of workforce, geographic barriers, and uneven availability of outpatient psychiatric services (19, 20). The Great Plains and Rocky Mountain areas, which include the states of Colorado, Kansas, Montana, Nebraska, New Mexico, North Dakota, Oklahoma, South Dakota, Texas, Wyoming, Idaho, and Utah have distinct geographical and socioeconomic contexts that may influence psychological results. These regions are distinguished by large rural areas (21) and limited access to healthcare resources. Rural populations face unique challenges such as higher rates of poverty, restricted access to healthcare, low health literacy, and social isolation, all of which can exacerbate mental health issues (22–25). Monnat and Pickett (26) reported significant rural–urban differences in mental health outcomes, while Andrilla et al. (27) documented substantial shortages of mental health providers across rural America. Similarly, Konrad et al. (28) identified large geographic disparities in access to psychiatric care, particularly in sparsely populated regions. These challenges are especially pronounced in the Great Plains and Rocky Mountain regions, where large rural populations and limited healthcare infrastructure may contribute to elevated mental health vulnerability. This demonstrates that regional studies are critical for understanding and alleviating mental health inequities since the mental health crisis is compounded in some areas by more severe barriers to treatment, high expenses, and a scarcity of mental health experts (29).

Global statistical models employed in public health research frequently fail to account for spatial non-stationarity and autocorrelation, resulting in potentially misleading findings. County-level mental health research has increasingly shown that geographic context matters. Lee et al. (5) demonstrated that the relationship between mentally unhealthy days and social vulnerability varies considerably across geographic regions, indicating that determinants of mental health are spatially non-stationary rather than geographically uniform. Counties characterized by socioeconomic disadvantage, housing instability, transportation barriers, and demographic vulnerability experienced different levels of mental health burden depending on their geographic context. Evidence further indicates that mental health outcomes are influenced not only by local conditions but also by broader regional contexts. Yang et al. (6) examined county-level mental distress and showed that both within-county and between-county processes contribute to mental health outcomes. Their results highlighted the importance of spatial dependence, demonstrating that characteristics of neighboring counties can influence local levels of mental distress.

Geographically weighted regression (GWR) is one of the strategies that solve constraints by studying local variations and the associations between health outcomes and other socioeconomic and demographic factors (5, 30). Ha (31) explored the use of GWR in health research, emphasizing its benefits over typical global models. The study showed how GWR might reveal large local changes that global models miss. For example, the association between mentally unwell days and socioeconomic level differed among regions. Building on this body of evidence, we examine how county-level socioeconomic conditions, healthcare access, and demographic composition are associated with frequent mental distress across different geographic locations, and whether these associations vary across space and time. To address this gap, we incorporated the multiscale geographically and temporally weighted regression (MGTWR) to account for these variations and provided a comparative analysis with global ordinary least squares (OLS) models and GTWR. The approach aimed to offer a more nuanced understanding of the spatio temporal dynamics influencing mental health, providing valuable insights for health governance and targeted interventions, while recognizing that the study is observational and ecological. Therefore, the findings are interpreted as county-level associations rather than causal effects.

The main objectives of this study were:

- What socio-demographic and economic factors are associated with the spatial variation of FMD in the Great Plains and Rocky Mountains?
- What are the geographical mental health trends in the Great Plains and Rocky Mountains?

The rest of the paper is organized as follows: The methodology section gives an overview of the methods used in the analyses, including a description of the data; the results section describes the results of the study. Lastly the Discussion and Conclusion presents the discussion and conclusion of the study.

## 2 Methodology

This section details the study area and the data used; the data was obtained from the County Health Rankings and Roadmaps (32) for the dependent and some of the independent variables. The section also feature the statistical methods such as the Moran's I test, used to identify spatial autocorrelation in the data and the regression models.

### 2.1 Study area

Mental health issues are more prevalent in rural areas of the USA, largely due to significant barriers to accessing mental health services, such as limited availability of qualified providers, high treatment costs, and social stigma (33). Recognizing these challenges, our study is focused on regions with expansive rural populations, particularly the Great Plains and Rocky Mountains states of the United States of America, where these barriers are most pronounced (34). The study region spans the Great Plains and Rocky Mountain counties of the states Colorado, Kansas, Montana, Nebraska, New Mexico, North Dakota, Oklahoma, South Dakota, Texas, Wyoming, Idaho, and Utah in the United States America.

#### 2.1.1 Great plains

**Figure 1:**
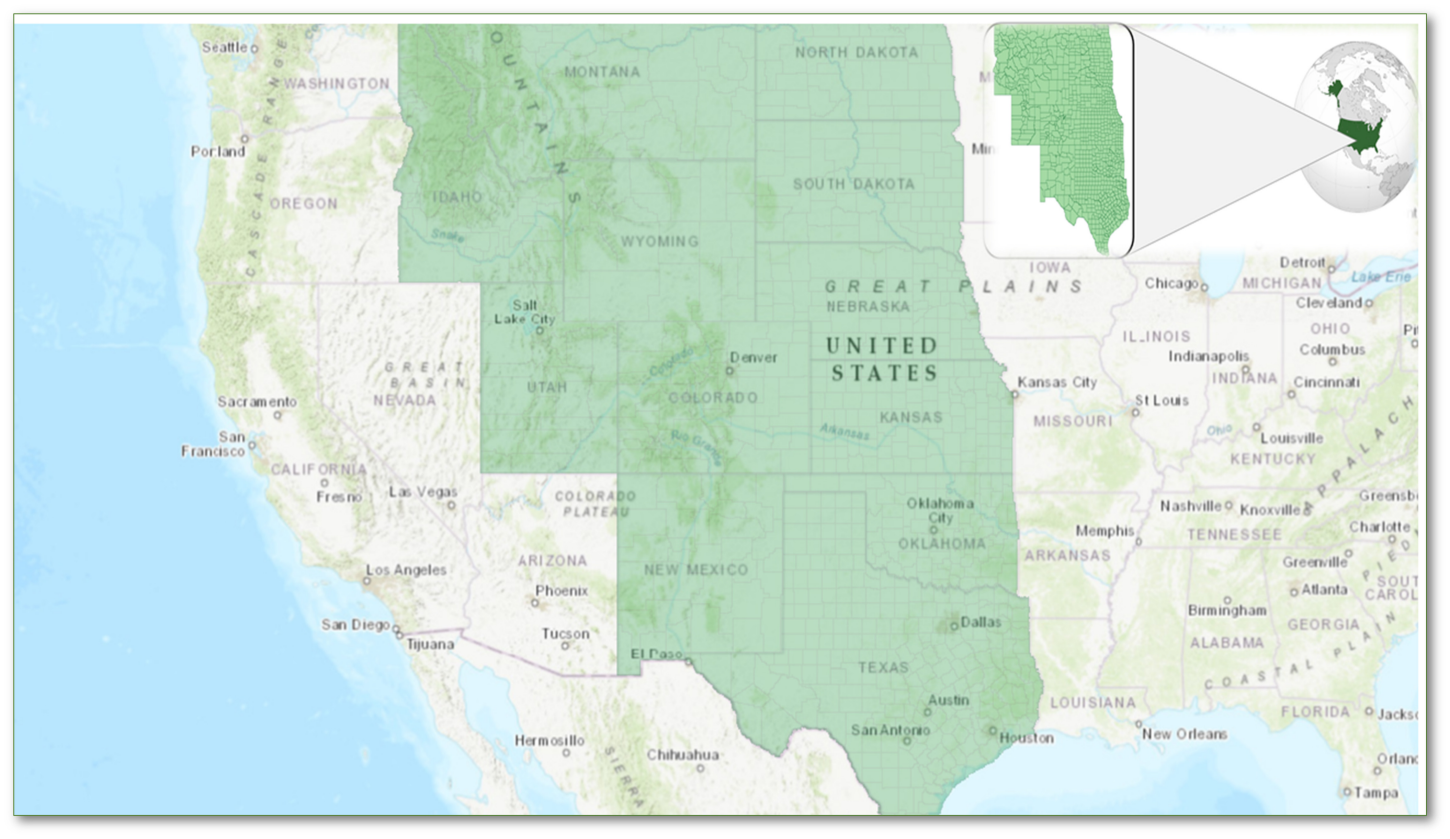
Study area spanning the Great Plains and Rocky Mountain regions.

The Great Plains is a broad expanse of flat land in North America, covered in prairie, steppe, and grassland, see Figure 1. It spans the United States and Canada, covering parts of ten U.S. states (Montana, North Dakota, South Dakota, Wyoming, Nebraska, Kansas, Colorado, Oklahoma, Texas, and New Mexico) and three Canadian provinces (Alberta, Saskatchewan, and Manitoba). They extend from latitudes 28° to 49° north and longitudes 97° to 114° west, encompassing an area of approximately 1,300,000 square kilometers (35). The topography of the Great Plains is characterized by a flat landscape, hilly areas and river valleys. The climate varies significantly from north to south and east to west. Generally, it experiences a continental climate with hot summers and cold winters (36).

#### 2.1.2 Rocky mountains

The Rocky Mountains as shown in Figure 1 are a major mountain range in western North America, extending from the northernmost part of British Columbia in Canada through the U.S. states of Idaho, Montana, Wyoming, Utah, Colorado, and New Mexico, spanning approximately 4,800 kilometers. The range spans latitudes from about 32° to 53° north and longitudes from 105° to 125° west (37). They feature diverse topography, including rugged mountains, high plains, and deep valleys. The highest peak in the range is Mount Elbert in Colorado, which stands at 4,401 meters above sea level. The climate varies widely depending on altitude and latitude. .

### 2.2 Dataset

The data sources for this study included shapefile data obtained from the United States Census Bureau for regions in the Great Plains and the Rocky Mountains (38) and health data sourced from the County Health Rankings and Roadmaps website (32). Shapefile data was joined with CSV health data using QGIS Desktop 3.36.3 (39), linking geographical information to health data for spatial analysis. The final dataset consisted of 897 counties observed annually from 2019 to 2023, resulting in 4,485 county-year observations. No missing values were present in the final merged dataset; therefore, no imputation or listwise deletion was required.

The analysis focused on the following variables: the dependent variable being the frequent mental distress, which is the percentage of adults reporting 14 or more days of poor mental health per month (45), and the independent variables included the education level, poverty, race, age, percentage of uninsured adults, percentage of population living in a rural area, percentage female, and unemployment rate as given in Table 1. The selected independent variables were chosen to capture three key domains: socioeconomic conditions, healthcare access, and demographic composition. Socioeconomic conditions were represented by unemployment rate, poverty rate, and educational attainment since they have been consistently linked to poor mental health outcomes (46, 47). Healthcare access was represented by the percentage of uninsured individuals as availability of mental health services represents a critical structural determinant of mental health disparities (19, 20, 28). This was done recognizing that insurance status captures only one dimension of access to care. Demographic composition was represented by the population over 65 years, female population, and racial/ethnic composition, which have been shown to shape patterns of mental health and access to care across populations (48–50).

**Table 1:** Description of outcome and explanatory variables and data sources.

Parameter	Description	Source
Frequent mental distress	Percentage of adults reporting 14 or more days of poor mental health per month.	Behavioral Risk Factor Surveillance System (40)
Poverty rate	Percentage of individuals living below the federal poverty level.	American Community Survey (41)
Population aged 65+	Percentage of the population aged 65 years and older.	Census Population Estimates (42)
Race/ethnicity	Population percentages of racial groups, including American Indian, Asian, Black, Native Hawaiian, and Hispanic populations. The non-Hispanic White population was excluded and used as the reference category.	Census Population Estimates (42)
Gender (% female)	Percentage of females in the population.	Census Population Estimates (42)
Rural area percentage	Percentage of the population living in census-defined rural areas.	Census Population Estimates (42)
Unemployment rate	Percentage of the population aged 16 years and older who are unemployed but seeking work.	Behavioral Risk Factor Surveillance System (40)
% Uninsured	Percentage of the population under age 65 without health insurance.	U.S. Census Bureau–American Community Survey (43)
% With less than high school diploma	Percentage of adults aged 25 years and older whose highest level of education is 9th to 12th grade, without a high school diploma.	U.S. Census Bureau–American Community Survey (44)
% With bachelor's degree or higher	Percentage of adults aged 25 years and older with a bachelor's degree or higher.	U.S. Census Bureau–American Community Survey (44)

The variables were also used to represent both contextual and compositional characteristics of counties. Contextual variables, such as unemployment, poverty, and insurance coverage, reflect structural and socioeconomic conditions that may be associated with mental health outcomes. In contrast, demographic variables, including age structure, gender distribution, and racial/ethnic composition, are compositional measures that describe the population structure of each county rather than direct exposures. As such, the inclusion of both types of variables allowed for a more comprehensive assessment of geographic variation in FMD, while recognizing that compositional variables should be interpreted as area-level characteristics rather than causal determinants. In this study, all variables are measured at the county level. As such, the analysis captures associations between population-level characteristics and frequent mental distress rather than individual-level relationships.

### 2.3 Methods used

#### 2.3.1 Moran's I

Global and local Moran's I statistics were used to assess spatial autocorrelation and clustering patterns of FMD across the study region. The global Moran's I measures the overall degree of spatial autocorrelation, with values ranging from −1 (perfect dispersion) to 1 (perfect clustering), and a value of 0 indicating spatial randomness. Moran's I (40) is calculated as follows:


$$\begin{array}{l} I=\frac{N\overset{N}{\underset{i=1}{\sum }}\overset{N}{\underset{j=1}{\sum }}w_{ij}\left(x_{i}-\bar{x}\right)\left(x_{j}-\bar{x}\right)}{S_{0}\overset{N}{\underset{i=1}{\sum }}{\left(x_{i}-\bar{x}\right)}^{2}} \end{array}$$ (1)

where *N* is the number of observations; *x*_*i*_ and *x*_*j*_ are the values at locations *i* and *j*; $\bar{x}$ is the mean; *w*_*ij*_ is the spatial weight between observations; and *S*_0_ is the sum of all spatial weights.

Local Moran's I was used to identify spatial clusters and outliers, enabling the detection of high–high (hotspots), low–low (coldspots), and spatial outliers across counties. Spatial weights were defined using a k-nearest neighbors approach (*k* = 10) combined with an inverse distance weighting of 2, ensuring that each county was compared with a consistent number of nearby counties.

#### 2.3.2 Ordinary least squares

To estimate the average county-level associations between frequent FMD and the selected explanatory variables, we first applied an ordinary least squares (OLS) model. This model served as the global baseline against which the performance of the geographically weighted models (GTWR and MGTWR) was evaluated.

The OLS model (51) was calculated as:


$$\begin{array}{l} y=\beta_{0}+\overset{m}{\underset{k=1}{\sum }}\beta_{k}x_{k}+\epsilon_{i} \end{array}$$ (2)

where *y* denotes the dependent variable, *x*_*k*_ represents the *k*-th explanatory variable, *m* is the number of predictors, β_0_ is the intercept, β_*k*_ are the regression coefficients, and ϵ_*i*_ is the error term.

#### 2.3.3 Geographically and temporally weighted regression (GTWR)

The geographically and temporally weighted regression (GTWR) model (52) can be expressed as follows:


$$\begin{array}{l} Y_{i}=\beta_{0}\left(u_{i},v_{i},t_{i}\right)+\overset{m}{\underset{k=1}{\sum }}\beta_{k}\left(u_{i},v_{i},t_{i}\right)X_{ik}+\epsilon_{i} \end{array}$$ (3)

where *Y*_*i*_ and *X*_*ik*_ are the response variable and the explanatory variables at each space-time location *i* (*i* = 1, 2, ..., *n*), respectively. β_0_(*u*_*i*_, *v*_*i*_, *t*_*i*_) is the constant term for location *i*, and β_*k*_(*u*_*i*_, *v*_*i*_, *t*_*i*_) is the regression coefficient of the *k*-th explanatory variable at point *i*. The estimation of β_*k*_(*u*_*i*_, *v*_*i*_, *t*_*i*_) can be expressed as


$$\begin{array}{l} \beta \left(u_{i},v_{i},t_{i}\right)={\left(X^{T}W\left(u_{i},v_{i},t_{i}\right)X\right)}^{-1}X^{T}W\left(u_{i},v_{i},t_{i}\right)Y \end{array}$$ (4)

where *W*(*u*_*i*_, *v*_*i*_, *t*_*i*_) = diag(α_*i*1_, α_*i*2_, ..., α_*in*_) is the spatiotemporal weight matrix, with *n* being the number of observations. The diagonal elements α_*ij*_ (1 ≤ *j* ≤ *n*) represent the space-time distance functions of (*u, v, t*) corresponding to the weights when calibrating a weighted regression near observation point *i*.

Given a spatial distance $d_{ij}^{S}$ and a temporal distance $d_{ij}^{T}$, the combined space-time distance $d_{ij}^{ST}$ is given by


$$\begin{array}{l} d_{ij}^{ST}=\lambda d_{ij}^{S}+\mu d_{ij}^{T} \end{array}$$ (5)

where


$$\begin{array}{l} {\left(d_{ij}^{S}\right)}^{2}={\left(u_{i}-u_{j}\right)}^{2}+{\left(v_{i}-v_{j}\right)}^{2},\;{\left(d_{ij}^{T}\right)}^{2}={\left(t_{i}-t_{j}\right)}^{2} \end{array}$$ (6)

Here, λ and μ are scaling factors to balance spatial and temporal distances. Using Euclidean distance and a Gaussian kernel to construct the space-time weight matrix, the weight function is given by:


$$\begin{array}{l} \alpha_{ij}=\exp\left(-\frac{{\left(d_{ij}^{ST}\right)}^{2}}{{\left(h^{ST}\right)}^{2}}\right) \end{array}$$ (7)

where *h*^*ST*^ is the spatiotemporal bandwidth parameter. The relationship between spatial and temporal bandwidths is expressed as:


$$\begin{array}{l} h_{S}^{2}=\frac{{\left(h^{ST}\right)}^{2}}{\lambda },\;h_{T}^{2}=\frac{{\left(h^{ST}\right)}^{2}}{\mu } \end{array}$$ (8)

where *h*_*S*_ and *h*_*T*_ are the spatial and temporal bandwidth parameters, respectively. Let τ denote the parameter ratio μ/λ. Setting λ = 1 simplifies the estimation process by reducing the number of parameters to be estimated in practice.

We applied GTWR to capture spatial and temporal non-stationarity in the associations between FMD and the covariates. A Gaussian kernel with an adaptive bandwidth specification was used, allowing the number of neighboring observations included in each local regression to remain constant while the spatial extent of the bandwidth varies across locations, since the study region includes both densely populated and sparsely populated counties. This ensured stable local estimates in areas with varying data density by adapting the bandwidth to local data structure, which reduced bias in sparsely populated regions while preserving sensitivity to local variation in more densely populated areas.

#### 2.3.4 Multiscale geographically and temporally weighted regression (MGTWR)

The multiscale geographically and temporally weighted regression (MGTWR) model, proposed by Wu et al. (53), extends the GTWR model by allowing each explanatory variable to have its own spatial and temporal bandwidths. It is expressed as follows:


$$\begin{array}{l} Y_{i}=\beta_{bwt_{0}-s_{0}}\left(u_{i},v_{i},t_{i}\right)+\overset{m}{\underset{k=1}{\sum }}\beta_{bwt_{k}-s_{k}}\left(u_{i},v_{i},t_{i}\right)X_{ik}+\epsilon_{i} \end{array}$$ (9)

where (*u*_*i*_, *v*_*i*_, *t*_*i*_) represents the space-time coordinates of observation *i* (*i* = 1, 2, …, *n*), with *t*_*i*_ as the observation time. *Y*_*i*_ is the dependent variable at location *i*, and *X*_*ik*_ is the *k*-th explanatory variable at location *i*. β_*bw*_*t*__0_−*s*_0__(*u*_*i*_, *v*_*i*_, *t*_*i*_) is the intercept at location *i*, while β_*bw*_*t*__*k*_−*s*_*k*__(*u*_*i*_, *v*_*i*_, *t*_*i*_) represents the regression coefficient of the *k*-th explanatory variable at location *i*.

Determinants of mental health, including socioeconomic conditions, access to care, and demographic composition, are likely to operate at different spatial and temporal scales. Unlike GTWR, which applies a single spatial and temporal bandwidth across all variables, MGTWR allows each covariate to vary at its own scale. This enables the model to distinguish localized effects from broader regional patterns, providing a more nuanced understanding of spatial heterogeneity in mental health outcomes.

#### 2.3.5 Model calibration

For the GTWR model, the optimal bandwidths were determined using the corrected Akaike Information Criterion (AICc). In contrast, the MGTWR model was calibrated using the back-fitting method based on maximum likelihood estimation (54). The iterative process continued until the change in the score (SOC) between successive iterations became sufficiently small. The gaussian kernel function and the adaptive bandwidth approach were used for both GTWR and MGTWR.

In this study, the convergence criterion for MGTWR calibration was set at


$$\begin{array}{l} \text{SOC}<10^{-2} \end{array}$$ (10)

Once this threshold was met, the iteration process was terminated, and the estimated coefficients from the final iteration were retained as the model's final estimates.

Additionally, the maximum spatial scale was initially estimated based on the geographic distribution of the study area, while the maximum temporal scale was defined by the duration of the data collection period which was yearly.

#### 2.3.6 Statistical analysis

First, global Moran's I (55) was used to assess the overall spatial autocorrelation of FMD across the study region. Moran's I scatter plots and local cluster maps were further examined in GeoDa (56) to identify localized spatial clustering patterns.

To investigate the associations between FMD and the selected county-level covariates, three regression approaches were applied: OLS, GTWR, and MGTWR. Model performance was evaluated using the coefficient of determination (*R*^2^), adjusted *R*^2^, Akaike Information Criterion corrected for small sample sizes (AICc), and root mean square error (RMSE). All basic statistical analyses were conducted in Python version 3.14.5 (Python Software Foundation, Beaverton, United States), and the models used were implemented using an open-source Python library available on GitHub. In particular, we used the multiscale geographically and temporally weighted regression (MGTWR) repository maintained by Sunkun (57).

## 3 Results

**Figure 2:**
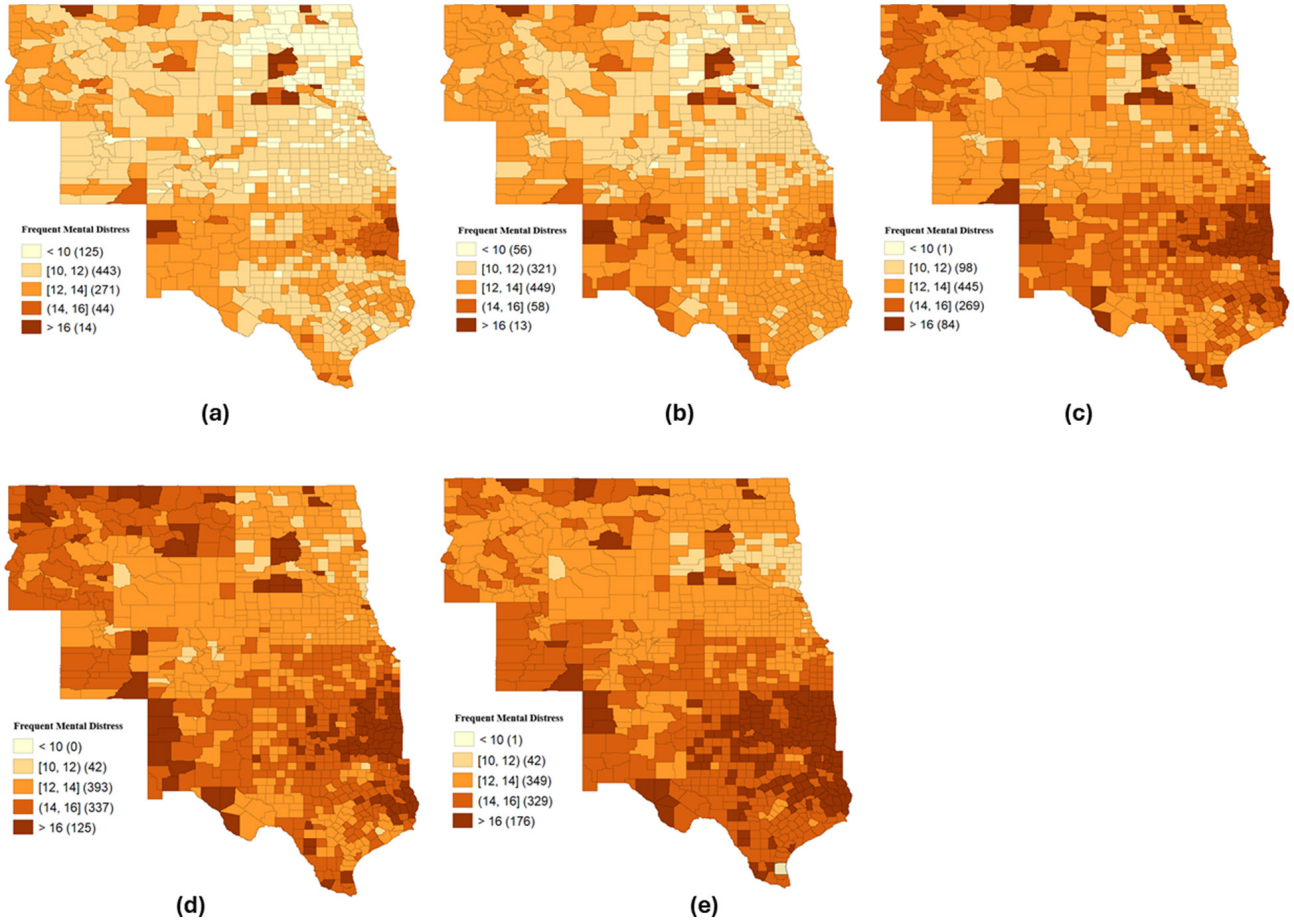
The spatial distribution pattern of frequent mental distress in the Great Plains and Rocky Mountains, 2019–2023. **(a)** FMD for the year 2019; **(b)** FMD for the year 2020; **(c)** FMD for the year 2021; **(d)** FMD for the year 2022; and **(e)** FMD for the year 2023.

The quantile maps for the study period, 2019 to 2023, in Figure 2 illustrated both temporal change and geographic patterning of FMD across the study region. They revealed a concerning upward trend in the percentage of adults reporting 14 or more days of poor mental health per month. The number of counties where 16% or more of adults reported FMD rose, from only 14 in 2019 to 176 in 2023. A particularly sharp increase occurred in 2021, when the number of counties exceeding this threshold jumped from 13 in the previous year to 84.

In addition to the temporal increase, the maps revealed pronounced spatial heterogeneity in FMD across the study region. Counties in the southern portion of the study area, particularly in Texas and Oklahoma, consistently exhibited higher levels of FMD throughout the study period, whereas counties in northern states such as North Dakota, Montana, and Wyoming generally displayed lower levels. These regional patterns persisted over time, indicating that FMD was not uniformly distributed across counties but instead followed a clear geographic structure.

### 3.1 Spatial autocorrelation

**Figure 3:**
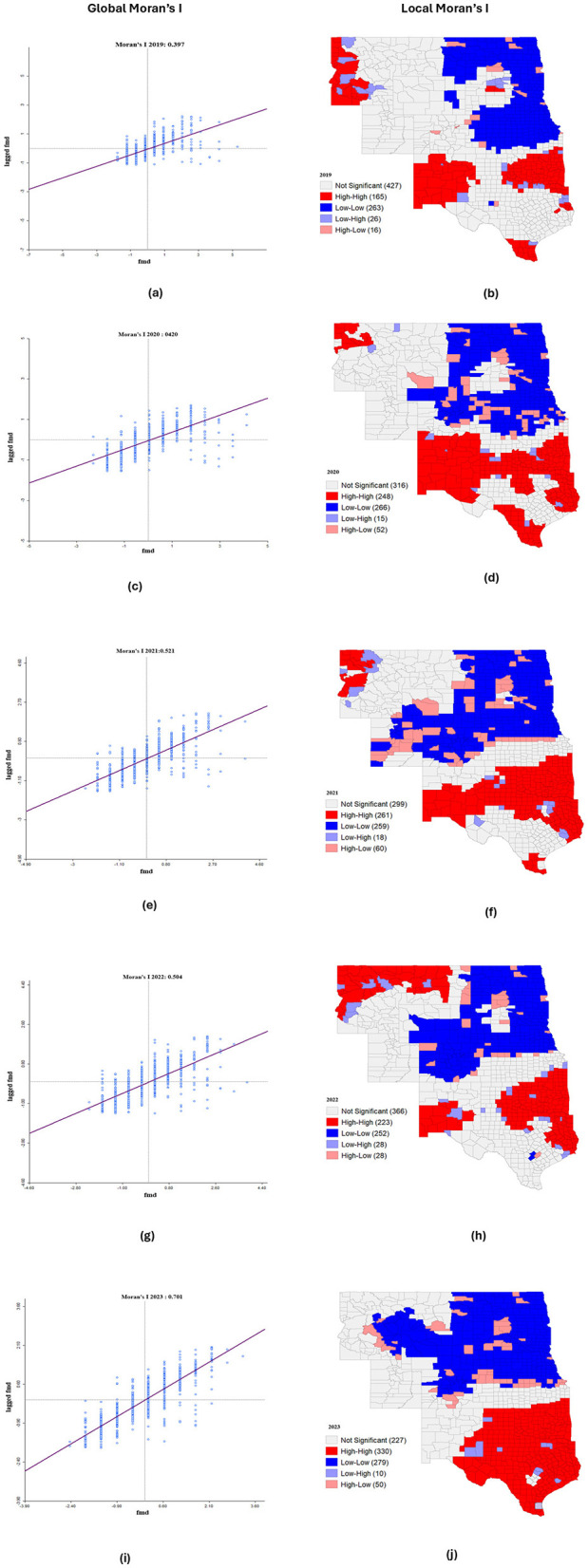
Moran's I statistics of frequent mental distress and corresponding cluster maps across the Great Plains and Rocky Mountains regions from 2019 to 2023. Each row corresponds to a year, with panels **(a, c, e, g, i)** showing FMD global Moran's I and **(b, d, f, h, j)** the associated spatial clusters.

To evaluate whether FMD exhibited spatial dependence across the Great Plains and Rocky Mountains from 2019 to 2023, both global and local Moran's I statistics were calculated (55, 58). The global Moran's I values shown in Figure 3 were consistently positive throughout the study period with the highest value being in 2023 (*I* = 0.701), indicating that counties with high levels of FMD tended to be geographically surrounded by counties with similarly high levels, while counties with low levels of FMD tended to cluster near other low-FMD counties rather than being randomly distributed across space.

The Moran's I scatter plots presented in Figures 3a, c, e, g, i further illustrated the presence of spatial dependence. In these plots, the slope of the regression line corresponds to the Moran's I coefficient and reflects the strength and direction of spatial autocorrelation. The increasingly steep positive slope observed over time indicates that spatial clustering in FMD strengthened progressively between 2019 and 2023.

Local Moran's I cluster maps were subsequently used to identify the geographic distribution of statistically significant local clusters of FMD. High–high clusters (hotspots) represented counties with elevated FMD values surrounded by neighboring counties that also had high FMD levels. In contrast, low–low clusters (coldspots) identified counties with low FMD values surrounded by similarly low neighboring counties. The local cluster maps also identified spatial outliers, including high–low clusters, where counties with high FMD were surrounded by low-FMD neighbors, and low–high clusters, where counties with low FMD were surrounded by high-FMD neighboring counties. These local indicators of spatial association provided insight into regional heterogeneity and localized departures from broader spatial trends.

In 2019, the high–high clusters were concentrated primarily in the central and southern regions of the study, while the cold spots were predominantly located in northern counties. Several isolated spatial outliers, represented by high–low and low–high clusters, were also observed in Figure 3b. By 2020, spatial clustering became more pronounced, with persistent hotspots remaining in the southern and central regions and coldspots continuing to dominate northern portions of the study area, as shown in Figure 3d.

In 2021, high–high clusters remained concentrated in the southern and central counties, although hotspot intensity decreased slightly in some southern areas compared with previous years, as illustrated in Figure 3f. Low–low clusters persisted across northern counties and expanded westward, while the frequency of spatial outliers increased in parts of the northern region. By 2022, hotspots became somewhat more spatially dispersed, and additional high–high clusters emerged in northwestern counties, as shown in Figure 3h. In 2023, strong hotspot clustering re-emerged in southern counties, whereas coldspot regions remained relatively stable compared with 2022, as presented in Figure 3j.

### 3.2 OLS results

To evaluate potential multicollinearity among the explanatory variables, the variance inflation factor (VIF) (59) was first calculated for all predictors included in the global regression model. The VIF is defined as


$$\begin{array}{l} {\text{VIF}}_{i}=\frac{1}{1-R_{i}^{2}} \end{array}$$ (11)

where $R_{i}^{2}$ represents the coefficient of determination obtained by regressing predictor *i* on the remaining explanatory variables. A VIF value exceeding 10 was interpreted as a considerable level of multicollinearity with other predictors, indicating that such variables may warrant removal or other approaches to deal with the multicollinearity concerns. In our analysis, none of the variables exceeded the value of 10, hence all the variables were retained (60). The resulting VIF values are presented in Table 2.

**Table 2:** Variance inflation factor (VIF) values for explanatory variables included in the regression models.

Variable	VIF
Unemployment rate	1.4081
65+ years	2.0492
Black population	1.5044
Hispanic population	2.1339
Asian population	1.4142
American Indian population	1.5119
Native Hawaiian population	1.1033
Female population	1.1000
Rural population	2.0751
No high school diploma	2.3453
Bachelor's degree or higher	1.2039
Percent uninsured	1.8237
Poverty rate	1.2207

To further assess model stability and evaluate the relative contribution of correlated socioeconomic variables, a least absolute shrinkage and selection operator (LASSO) regression with cross-validation was performed (61) as shown in Table 3.

**Table 3:** LASSO regression coefficients.

Variable	LASSO coefficient
Unemployment rate	0.2373
65+ years	0.1337
Black population	0.0739
Hispanic population	–0.0153
Native Hawaiian population	0.0983
Asian Population	–0.0582
American Indian population	0.3054
Female population	0.0162
Population in rural	–0.0416
No High school diploma	0.0000
Bachelor's degree or higher	0.0397
Percent uninsured	0.3491
Poverty rate	0.0411

The optimal penalty parameter selected by cross-validation was α = 0.006764 with an RMSE of 0.7718.

The optimal penalty parameter selected through cross-validation was α = 0.006764, resulting in an RMSE of 0.7718. All explanatory variables were retained by the LASSO model except the individuals without a high school diploma, which was shrunk completely to zero, suggesting that it contributed limited independent explanatory value after accounting for he remaining socioeconomic indicators.

Table 4 presents the results of the OLS model. Several socioeconomic and demographic variables were significantly associated with variation in FMD across counties. Unemployment rate, proportion of the population aged 65 years and older, poverty rate, and percentage of uninsured individuals were all positively associated with higher levels of FMD.

**Table 4:** The coefficients and significance of the OLS.

Variable	Coef.	Sth. Err.	*t*	*P*-value
Poverty rate	0.0501^***^	0.013	3.933	0.000
Unemployment rate	0.2382^***^	0.014	17.408	0.000
65+ years	0.1531^***^	0.017	9.223	0.000
Black population	0.0819^***^	0.014	5.792	0.000
Hispanic population	–0.0313	0.017	–1.858	0.063
Asian population	–0.0709^***^	0.014	–5.169	0.000
American Indian population	0.3134^***^	0.014	22.099	0.000
Native Hawaiian population	0.1052^***^	0.012	8.680	0.000
Female population	0.0181	0.012	1.500	0.134
Population in rural	–0.0666^***^	0.017	–4.007	0.000
Bachelor's degree or higher	0.0512^***^	0.013	4.048	0.000
Percent uninsured	0.3635^***^	0.016	23.341	0.000

^*^p < 0.05, ^**^p < 0.01, ^***^p < 0.001.

Racial and ethnic composition variables also exhibited significant associations relative to the reference category (non-Hispanic White population). Counties with higher proportions of Black, American Indian and Alaska Native, and Native Hawaiian populations were associated with higher levels of FMD, whereas higher proportions of Hispanic and Asian populations were associated with lower levels of FMD.

The proportion of the rural population was negatively associated with FMD, suggesting lower reported distress in more rural counties. The female population was not statistically significant in the global model.

Since the explanatory variables were derived from 5-year ACS estimates, a pooled county-level sensitivity analysis was conducted to assess the potential influence of temporal misalignment between annually measured health outcomes and ACS-based socioeconomic indicators which may introduce bias in spatiotemporal analyses if short-term fluctuations are interpreted as contemporaneous effects (62). ACS 5-year estimates provide more stable measures for sparsely populated counties but represent rolling averages rather than exact point-in-time values. To evaluate the robustness of the annual models, all variables were averaged across the 2019–2023 period and a pooled OLS model was estimated using county-level mean values. The results are presented in Table 5.

**Table 5:** Pooled county-level OLS results based on 2019–2023 averaged variables.

Variable	Coef.	Sth. Err.	*t*	*P*-value
Poverty	0.1414^***^	0.027	5.213	0.000
Unemployment rate	0.2202^***^	0.025	8.962	0.000
Population aged 65+	0.0466^*^	0.023	2.067	0.039
Black population	0.0658^**^	0.019	3.436	0.001
Hispanic population	–0.0668^**^	0.024	–2.821	0.005
Asian population	–0.0966^***^	0.018	–5.332	0.000
American Indian population	0.2648^***^	0.020	13.443	0.000
Native Hawaiian population	0.0728^***^	0.016	4.507	0.000
Female population	0.0588^***^	0.017	3.550	0.000
Rural population	–0.0399	0.022	–1.793	0.073
Bachelor's degree or higher	–0.0086	0.024	–0.352	0.725
Uninsured population	0.3399^***^	0.021	15.856	0.000

^*^p < 0.05, ^**^p < 0.01, ^***^p < 0.001.

The pooled model demonstrated improved fit relative to the annual OLS specification (*R*^2^ = 0.641, adjusted *R*^2^ = 0.636), suggesting that persistent county-level structural differences explained a substantial proportion of variation in FMD across the study region. Poverty, unemployment, and uninsured population remained significantly and positively associated with FMD, while demographic composition variables showed heterogeneous associations relative to the omitted reference category (non-Hispanic White population). Counties with larger American Indian populations exhibited the strongest positive association with FMD, whereas Hispanic and Asian populations generally showed negative associations.

Some variables displayed sensitivity to temporal aggregation. The female population variable became statistically significant in the pooled specification, whereas the proportion of residents with a bachelor's degree or higher was no longer significant after temporal averaging. Nevertheless, the overall consistency in coefficient direction and significance across the annual and pooled models suggests that the principal findings were robust to ACS timing effects and were driven largely by persistent structural inequalities between counties rather than short-term temporal fluctuations.

### 3.3 Sensitivity analysis using the pooled county-level OLS results

### 3.4 GTWR and MGTWR results

**Figure 4:**
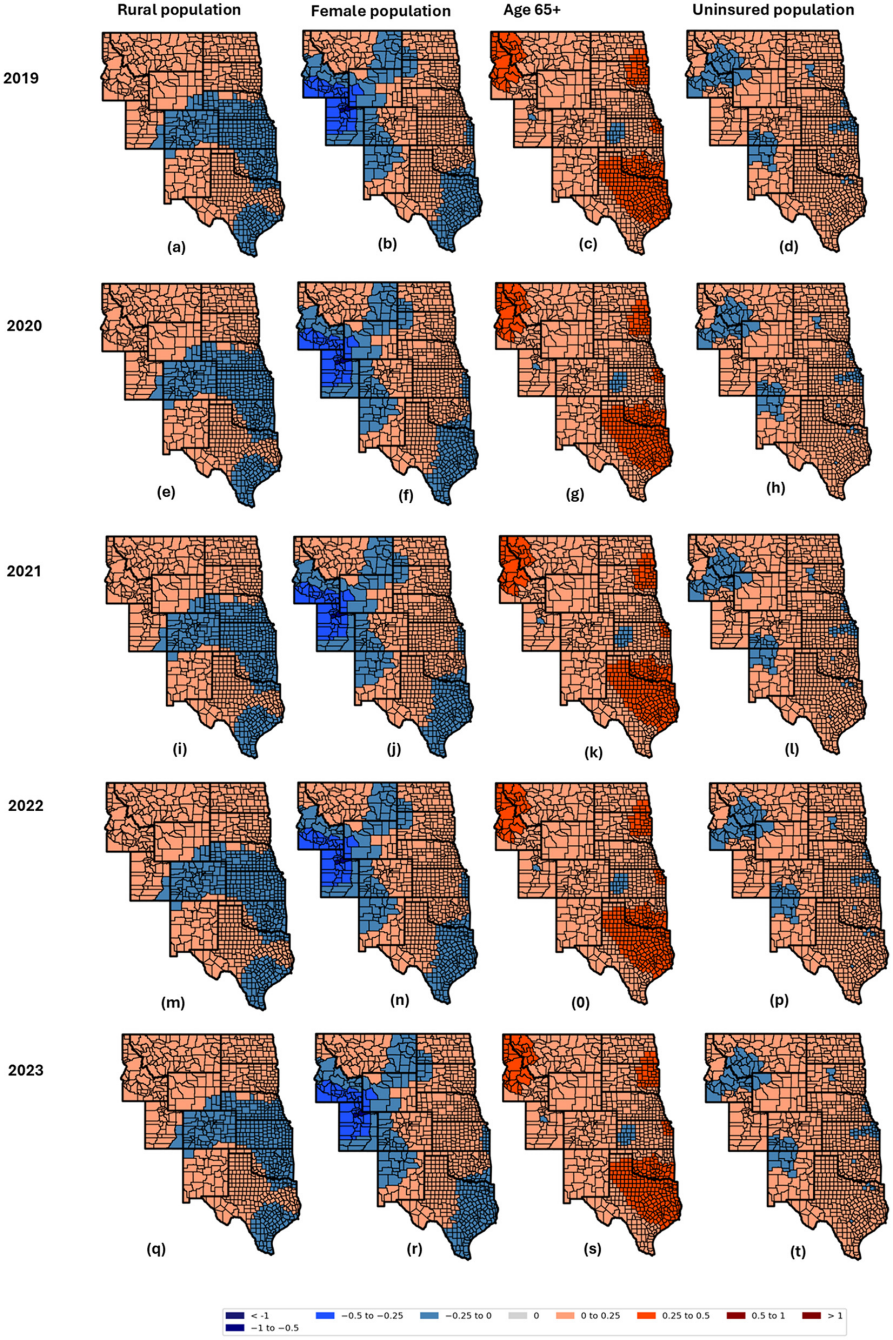
Spatial variation in MGTWR coefficients for demographic and health indicators from 2019 to 2023. **(a, e, i, m, q)** Rural population; **(b, f, j, n, r)** Female population; **(c, g, k, o, s)** Over age 65; **(d, h, l, p, t)** Uninsured. Red indicates positive associations with frequent mental distress; blue indicates negative associations, with darker tones reflecting stronger coefficient values.

**Figure 5:**
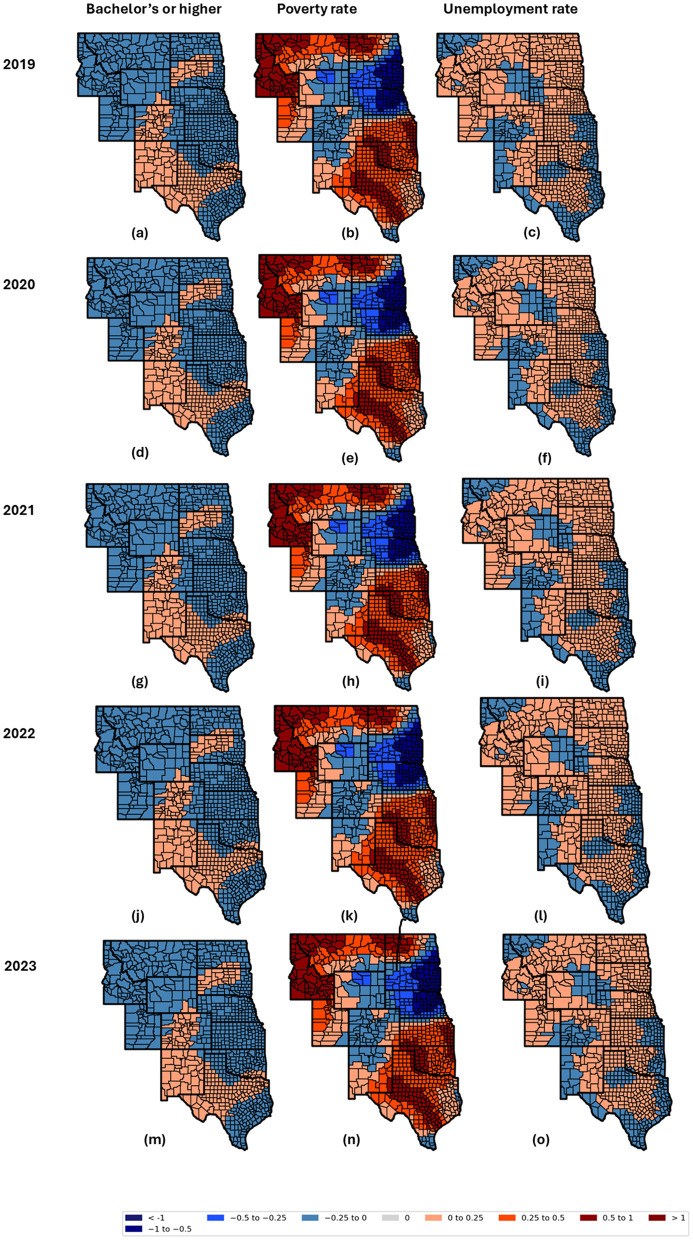
Spatial variation in MGTWR coefficients for education and socio-economic indicators from 2019 to 2023. **(a, d, g, j, m)** Bachelor's degree or higher, **(b, e, h, k, n)** poverty rate, and **(c, f, i, l, o)** unemployment rate. Red indicates positive and blue indicates negative associations with frequent mental distress.

**Figure 6:**
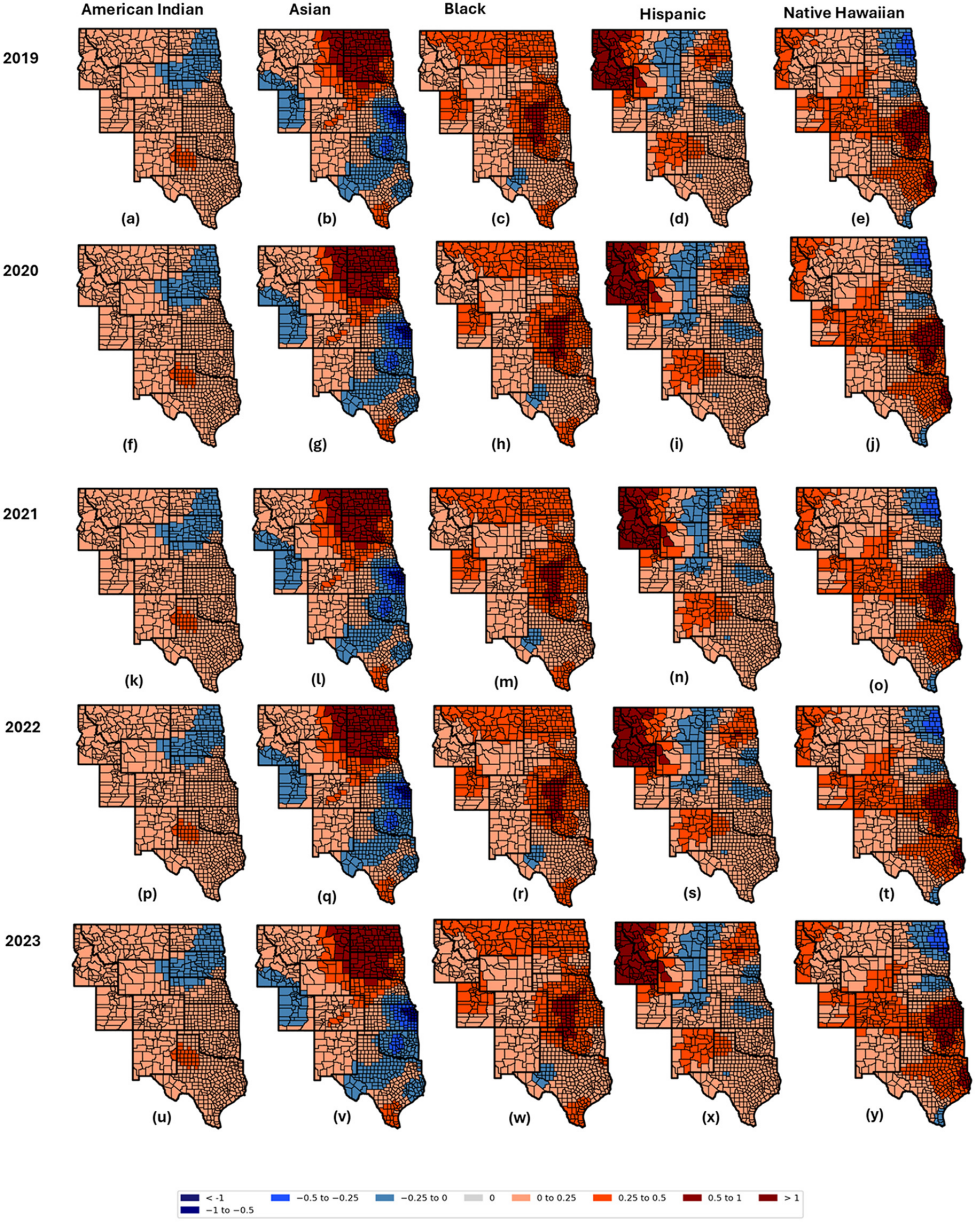
Spatial variation in MGTWR coefficients for racial and ethnic composition variables from 2019 to 2023. **(a–e)** show the American Indian, Asian, Black, Hispanic, and Native Hawaiian populations in 2019; **(f–j)** show the same variables in 2020; **(k–o)** in 2021; **(p–t)** in 2022; and **(u–y)** in 2023. Red shading indicates positive associations with frequent mental distress, whereas blue shading indicates negative associations, with darker tones reflecting stronger coefficient values. The non-Hispanic White population was used as the reference category.

The MGTWR results are presented in Figures 4–6. The coefficient maps were classified using a diverging color scale ranging from strong negative associations to strong positive associations. Coefficients < −1 represented strongly negative associations, while values from −0.5 to −0.25 and −0.25 to 0 represented moderate and weak negative associations, respectively. Values close to 0 indicated little or no local association. Positive coefficients ranging from 0 to 0.25 represented weak positive associations, values from 0.25 to 0.5 and 0.5 to 1 represented moderate to strong positive associations, and those >1 indicated strongly positive associations. The color scale ranged from dark blue for strong negative coefficients to dark red for strong positive coefficients, allowing visual comparison of the magnitude and direction of local associations across counties and years.

The rural population displayed heterogeneous associations with FMD across the study region, as illustrated in Figures 4a, e, i, m, q. Although several counties exhibited negative associations, particularly in Nebraska, Kansas, Colorado, Oklahoma, and Texas, other regions demonstrated positive coefficients, indicating that the relationship between rurality and FMD varied substantially across geographic contexts.

Similarly, the female population variable exhibited spatially varying effects across the study area, as shown in Figures 4b, f, j, n, r. Counties with higher proportions of female populations were associated with lower levels of FMD in several regions, while negative associations were particularly evident in parts of Utah, Idaho, Montana, New Mexico, and Texas.

In contrast, the population aged 65 years and older demonstrated predominantly positive associations with FMD throughout much of the Great Plains and Rocky Mountains during the study period, as illustrated in Figures 4c, g, k, o, s. Similarly, the proportion of uninsured individuals showed largely positive associations with FMD across most counties and years, indicating that reduced healthcare access was consistently associated with higher levels of mental distress.

Educational attainment and socioeconomic variables also exhibited substantial geographic heterogeneity, as shown in Figure 5. Counties with higher proportions of residents holding a bachelor's degree or higher generally demonstrated negative associations with FMD, although localized positive relationships were observed in portions of Texas, New Mexico, Wyoming, and North Dakota.

Poverty rates displayed mixed spatial effects across the study region. Higher poverty levels were generally associated with increased FMD, although several counties in North Dakota, South Dakota, Nebraska, and New Mexico exhibited negative associations, suggesting that the relationship between poverty and mental distress varied geographically.

The unemployment rate demonstrated predominantly positive associations with FMD across most counties, indicating that higher unemployment was generally associated with increased mental distress. However, localized negative coefficients were observed in parts of New Mexico, Colorado, South Dakota, and Utah, suggesting that regional contextual factors may moderate the relationship between unemployment and mental health outcomes.

Racial and ethnic composition variables also demonstrated substantial spatial heterogeneity, as shown in Figure 6. Counties with larger proportions of American Indian populations generally exhibited positive associations with FMD across much of the study region, although negative coefficients were observed in portions of North and South Dakota. The Asian population variable exhibited positive associations across several counties in the Dakotas, while weaker or negative associations were observed in portions of Utah, Idaho, Nebraska, Kansas, and Texas. Similarly, Black, Hispanic, and Native Hawaiian population variables generally demonstrated positive associations with FMD across most counties, although localized negative associations were observed in selected regions including Montana, Wyoming, South Dakota, and Nebraska.

Table 6 presents the spatial and temporal bandwidths estimated for the GTWR and MGTWR models. In the GTWR model, a single adaptive spatial bandwidth of 71.2 km and temporal bandwidth of 10.0893 were applied uniformly across all variables. In contrast, the MGTWR model produced variable-specific bandwidths, indicating substantial differences in the spatial and temporal scales at which predictors influenced FMD. Variables such as the female population and rural population exhibited relatively large bandwidths, suggesting broader regional effects and greater spatial stability, whereas variables such as the American Indian population, poverty rate, and uninsured population showed comparatively smaller bandwidths, indicating stronger local heterogeneity and more geographically localized relationships.

**Table 6:** Spatial and temporal bandwidths for GTWR and MGTWR models.

Factors	MGTWR		GTWR	
	*d*_*S*_ (km)	*d*_*T*_	*d*_*S*_ (km)	*d*_*T*_
Intercept	25.1	12.8760		
Poverty rate	52.1	26.7267	71.2	10.0893
Unemployment rate	82.2	42.1677		
65+ years	69.5	35.6527		
Black population	60.0	30.7794		
Hispanic population	44.5	22.8280		
Asian population	99.4	50.9911		
Female population	182.5	93.6205		
Rural population	124.3	63.7646		
Bachelor's or higher	57.2	29.3430		
Percent uninsured	45.4	23.2897		
Native Hawaiian population	81.0	41.5521		
American Indian population	40.5	20.7761		

We also compared the performance of the OLS, GTWR, and MGTWR models as shown in Table 7. Relative to the global OLS model, both geographically weighted approaches substantially improved model fit, indicating the importance of accounting for spatial and temporal non-stationarity in FMD associations. The GTWR model improved the explained variance from an adjusted *R*^2^ of 0.404 in the OLS model to 0.578, while reducing the AICc from 10423.189 to 9212.952 and decreasing the RMSE from 0.771 to 0.625. The MGTWR model produced slightly improved predictive performance compared with GTWR, with an *R*^2^ of 0.613, adjusted *R*^2^ of 0.578, and RMSE of 0.622. Although the GTWR model yielded a marginally lower AICc than MGTWR, both geographically weighted approaches substantially outperformed the global OLS model, supporting the presence of significant spatial and temporal heterogeneity in county-level FMD relationships.

**Table 7:** Performance evaluation of OLS, GTWR, and MGTWR models.

Model	*R* ^2^	Adjusted *R*^2^	AICc	RMSE
OLS	0.405	0.404	10423.189	0.771
GTWR	0.609	0.578	9212.952	0.6249
MGTWR	0.613	0.578	9283.471	0.6219

AICc represents the Akaike Information Criterion corrected for small sample sizes, and RMSE represents the root mean square error.

To evaluate whether the GTWR and MGTWR models adequately accounted for spatial dependence, spatial autocorrelation in the models' residuals was assessed using global Moran's I statistics. The residuals from the GTWR model exhibited weak but statistically significant positive spatial autocorrelation (Moran's I = 0.0522, *p* = 0.0090), indicating that some spatial dependence remained unexplained after model calibration. In contrast, the residuals from the MGTWR model showed no statistically significant spatial autocorrelation (Moran's I = –0.0088, *p* = 0.3700), suggesting that the MGTWR framework more effectively captured the underlying spatial structure in the data.

The results of the GTWR model are presented in Figures A1, B2, and C3 in the supporting information. Figure A1 illustrates the spatial and temporal associations of demographic and health indicators, including rural population, female population, population aged 65 years and older, and uninsured population with FMD.

Overall, the GTWR coefficient maps showed patterns broadly similar to those observed in the MGTWR results. The association between rural population and FMD was heterogeneous across the Great Plains and Rocky Mountains. Positive local coefficients were observed across several regions, whereas negative associations were concentrated in counties within North Dakota, Montana, Texas, and large portions of Nebraska, Kansas, Colorado, and Oklahoma. The female population variable exhibited predominantly negative associations with FMD across much of the study region, although localized positive coefficients emerged in parts of Montana, Nebraska, Kansas, South Dakota, and Texas. In contrast, the proportion of residents aged 65 years and older and the uninsured population showed predominantly positive associations with FMD throughout most counties and study years.

Figure B2 presents the education and socioeconomic indicators, namely the proportion of residents with a bachelor's degree or higher, poverty rate, and unemployment rate.

The coefficient maps also revealed substantial spatial heterogeneity. The proportion of residents with a bachelor's degree or higher was mostly negatively associated with FMD, suggesting lower levels of FMD in counties with higher educational attainment in many areas. However, positive associations were observed in Wyoming, New Mexico, and selected counties in South Dakota and Texas. Poverty rate displayed clear geographic variation: stronger positive associations were observed in several southern and northern counties, whereas negative associations appeared across portions of the central study region. Unemployment rate was predominantly positively associated with FMD across the Great Plains and Rocky Mountains, indicating that counties with higher unemployment generally experienced higher levels of FMD.

Finally, Figure C3 displays the racial and ethnic composition variables, including Asian, Hispanic, Black, American Indian, and Native Hawaiian populations spatial associations with FMD relative to the non-Hispanic White reference category. Most racial and ethnic composition variables showed positive associations across large portions of the study region, although the magnitude varied from weakly positive to strongly positive. Native Hawaiian population shares exhibited the strongest positive local coefficients. The Asian population variable showed greater spatial heterogeneity, with negative associations in selected counties in Texas, Nebraska, Idaho, and Utah, and positive associations elsewhere. The Hispanic population variable showed negative associations in parts of Wyoming and Montana, while positive associations were observed across much of the remaining study region. The Black population variable was positively associated with FMD across most counties and years.

Across all variables, spatial variability was consistently pronounced, whereas temporal variation was comparatively limited, indicating that geographic differences played a more dominant role than temporal changes in shaping patterns of frequent mental distress across the Great Plains and Rocky Mountains.

Summary statistics of the local GTWR and MGTWR coefficients are presented in Appendix Tables 9, 10, respectively. These tables further demonstrate the presence of spatial non-stationarity in the relationships between frequent mental distress and the explanatory variables. Substantial variability was observed in the magnitude and direction of several local coefficients across counties, as reflected by the wide ranges between minimum and maximum values.

The GTWR coefficients generally exhibited greater dispersion and variability compared with the MGTWR coefficients, indicating that the multiscale framework of MGTWR produced more stable local parameter estimates across space and time. Variables such as unemployment, uninsured population, Hispanic population, Black population, and American Indian population displayed particularly strong geographic heterogeneity, with some counties exhibiting positive associations and others negative associations.

Overall, spatial variability was consistently pronounced across most variables, whereas temporal variation was comparatively limited, indicating that geographic differences played a stronger role than year-to-year temporal changes in shaping county-level patterns of FMD.

## 4 Discussion and conclusion

### 4.1 Discussion

Mental health remains a major public health challenge in the United States, particularly within rural and underserved regions where socioeconomic disadvantage, healthcare shortages, and geographic isolation intersect. Using county-level data from 2019 to 2023, this study examined the spatiotemporal distribution of frequent mental distress (FMD) across the Great Plains and Rocky Mountain regions and evaluated how demographic, socioeconomic, and healthcare-related factors were associated with mental health outcomes. Consistent with previous county-level studies (5, 6), the findings revealed substantial spatial heterogeneity in the determinants of FMD, indicating that relationships observed at the regional level often differed considerably across counties. Although FMD increased during the study period, spatial variation consistently exceeded temporal variation, suggesting that persistent geographic and structural inequalities exerted a stronger influence on mental health outcomes than short-term temporal fluctuations.

The spatial clustering identified through Moran's I analyses demonstrated that counties with similar levels of FMD were geographically concentrated rather than randomly distributed. Persistent hotspots were observed across portions of Texas, Oklahoma, Kansas, and Nebraska, whereas lower levels of distress were more common in northern portions of the study region. These findings align with Lee et al. (5), who demonstrated that mental health outcomes vary substantially across geographic contexts, and with Yang et al. (6), who highlighted the importance of contextual and neighboring-county influences on mental distress. Together, these studies suggest that mental health outcomes are embedded within broader social and geographic systems and that local conditions cannot be fully understood in isolation from surrounding environments.

The superior performance of GTWR and MGTWR relative to the global OLS model further emphasized the importance of accounting for spatial non-stationarity. Several variables that appeared weak or nonsignificant in the global model exhibited strong localized relationships in specific counties. This finding reinforces growing evidence that mental health determinants operate differently across places and that geographically uniform policy approaches may overlook important local needs. The observed spatial heterogeneity suggests that interventions designed using national or regional averages may be less effective than strategies tailored to local socioeconomic and healthcare contexts.

The study period coincided with the COVID-19 pandemic, a period characterized by widespread social, economic, and healthcare disruption. Previous research documented substantial increases in depression, psychological distress, loneliness, anxiety, substance use, and suicidal ideation among U.S. adults during the pandemic (9–12). The increasing prevalence of FMD observed during the study period is therefore consistent with broader national evidence demonstrating deterioration in psychological well-being during and following the pandemic. However, the relatively stable spatial patterns observed in the geographically weighted models suggest that the pandemic may have intensified existing vulnerabilities rather than fundamentally altering the geographic organization of mental health disparities.

Socioeconomic disadvantage emerged as one of the most consistent predictors of FMD. Counties characterized by higher poverty rates, unemployment, and uninsured populations generally exhibited higher levels of mental distress. These findings are consistent with extensive evidence linking economic insecurity to chronic stress exposure, financial strain, housing instability, reduced healthcare utilization, and poorer mental health outcomes (46, 63, 64). Importantly, the geographically weighted models revealed that the magnitude of these associations varied considerably across counties, suggesting that socioeconomic disadvantage interacts with local conditions such as labor market opportunities, social services, healthcare infrastructure, and community resources. Consequently, the mental health consequences of poverty and unemployment are unlikely to be uniform across geographic settings.

The positive association between uninsured population and FMD highlights the importance of healthcare accessibility in shaping county-level mental health disparities. Nevertheless, insurance coverage should be interpreted as a proxy for healthcare access rather than a direct measure of mental healthcare availability. County-level studies have documented substantial geographic disparities in mental health workforce capacity and psychiatric service availability across the United States. Konrad et al. (28) and Thomas et al. (20) demonstrated widespread shortages of mental health professionals, particularly in rural counties, while Sun et al. (19) reported low availability, long wait times, and marked geographic disparities in psychiatric outpatient services nationwide. These barriers are particularly relevant within the Great Plains and Rocky Mountain regions, where long travel distances, workforce shortages, and limited service availability may restrict access to care even among insured populations. Consequently, the observed associations likely reflect broader structural barriers to mental healthcare rather than insurance status alone.

Rurality exhibited substantial spatial heterogeneity across the study region. Although rural communities are often characterized by provider shortages, transportation barriers, and reduced healthcare accessibility, they may also benefit from stronger social cohesion and community connectedness. The coexistence of positive and negative local associations suggests that rurality should not be viewed as a universally protective or harmful characteristic. Instead, rural mental health outcomes likely reflect complex interactions among healthcare access, economic opportunity, social support networks, stigma, and local community resources. These findings further support the need for place-based approaches that recognize the diversity of rural experiences rather than treating rural populations as a homogeneous group.

Counties with larger populations aged 65 years and older generally exhibited higher levels of FMD. Older adults may be particularly vulnerable to loneliness, chronic disease burden, mobility limitations, bereavement, and social isolation, all of which have been linked to poorer mental health outcomes (65, 66). The persistence of positive associations across both global and local models suggests that population aging remains an important dimension of mental health vulnerability within rural and underserved regions.

Educational attainment demonstrated considerable spatial heterogeneity. Although the global model identified a positive association between bachelor's degree attainment and FMD, local estimates varied substantially and were negative in many counties. Education may influence mental health through multiple pathways, including improved employment opportunities, greater health literacy, stronger coping resources, and enhanced access to healthcare. At the same time, highly educated populations may be more likely to recognize, report, and seek care for mental health concerns. The contrasting global and local findings therefore suggest that the relationship between education and mental health is context dependent and may differ according to local economic and social environments.

The racial and ethnic composition variables also demonstrated substantial geographic variation. Because these variables were modeled as population shares, the coefficients should be interpreted relative to the reference category, the non-Hispanic White population. Consequently, the observed associations reflect county-level compositional differences rather than individual-level mental health outcomes and should not be interpreted as causal effects of race or ethnicity. Instead, these patterns likely capture differences in exposure to social, economic, and structural conditions that influence mental health across communities.

Counties with larger Black population shares generally exhibited higher levels of FMD across much of the study region. Previous research has documented persistent disparities in psychological distress, depression, and access to mental healthcare among Black populations in the United States (67, 68). Structural racism, residential segregation, chronic stress exposure, economic disadvantage, and unequal access to healthcare have all been identified as important contributors to these disparities (69, 70). These findings should therefore be interpreted as reflecting broader structural inequalities and contextual disadvantages rather than inherent differences between racial groups.

Counties with larger American Indian and Native Hawaiian population shares also tended to exhibit higher levels of FMD across several portions of the study region. Indigenous communities frequently experience overlapping challenges related to historical trauma, poverty, geographic isolation, and limited access to behavioral healthcare services (17). Cromer et al. (16) further highlighted the substantial barriers to healthcare access facing American Indian and Alaska Native populations in rural areas, including transportation challenges, provider shortages, and limited availability of culturally appropriate services. These findings underscore the importance of culturally responsive approaches to mental healthcare that recognize the historical, social, and geographic contexts shaping Indigenous well-being.

The observed geographic variation across racial and ethnic groups may also reflect differences in social support systems, stigma, family processes, and healthcare experiences. Ghosh (71) emphasized the importance of family acceptance and psychosocial support in shaping mental health outcomes among marginalized populations, while Ghosh (72) highlighted the role of cultural competence, provider training, and affirming healthcare environments in reducing barriers to care. Together, these studies suggest that improving mental health outcomes requires more than expanding service availability alone; interventions must also address stigma, strengthen social support networks, and ensure that care is culturally responsive to the needs of diverse populations.

A key finding was that spatial variability consistently exceeded temporal variability across most explanatory variables. Although FMD increased over the study period, the geographically weighted models indicated that the spatial organization of mental health disparities remained comparatively stable over time. This suggests that persistent structural and geographic inequalities may exert a stronger influence on county-level mental health outcomes than short-term temporal changes. The results therefore emphasize the importance of place-based public health strategies that account for local socioeconomic and healthcare conditions rather than relying solely on broad regional or national interventions.

### 4.2 Conclusions

This study revealed pronounced spatial heterogeneity in frequent mental distress (FMD) across counties in the Great Plains and Rocky Mountains between 2019 and 2023. By applying geographically and temporally weighted regression (GTWR) and multiscale geographically and temporally weighted regression (MGTWR), we demonstrated that the associations between FMD and socioeconomic, demographic, and healthcare-related characteristics varied substantially across geographic space. Compared with the global OLS framework, the geographically weighted approaches provided improved representation of localized relationships and more effectively captured the spatial complexity underlying mental health disparities across the study region.

The findings indicate that mental health disparities across the Great Plains and Rocky Mountains are shaped by geographically uneven structural and socioeconomic conditions rather than uniform regional processes. In particular, unemployment, poverty, uninsured population, and aging populations exhibited predominantly positive associations with FMD across much of the study region, although the magnitude and direction of these relationships varied considerably between counties. Spatial variability consistently exceeded temporal variability, suggesting that geographic context played a more dominant role than short-term temporal change in shaping patterns of mental distress during the study period.

This study also highlights the importance of incorporating spatial non-stationarity into public health analyses. The persistence of spatial clustering and the substantial improvement of GTWR and MGTWR over global regression models emphasize that geographically varying relationships may be obscured when spatial dependence is ignored. These findings reinforce the need for place-based mental health strategies that account for local socioeconomic and healthcare conditions rather than relying solely on broad regional or national interventions.

Several limitations should be considered when interpreting these findings. The frequent mental FMD estimates used in this study were derived from modeled county-level estimates rather than direct measurements (73) and may therefore be less stable in sparsely populated counties or areas with limited survey representation. Consequently, uncertainty in the estimates may vary across counties, particularly within highly rural regions. Because the analysis was conducted at the county level, the observed relationships represent ecological associations and should not be interpreted as individual-level causal effects. In addition, the study is subject to the modifiable areal unit problem (MAUP), whereby observed relationships may vary according to the spatial scale of analysis. Although counties provide an appropriate balance between spatial resolution and data availability, alternative geographic units such as states or ZIP codes may yield different patterns. Furthermore, several explanatory variables were derived from five-year American Community Survey estimates, which may smooth short-term temporal variation, particularly in sparsely populated counties.

Future research should incorporate additional measures of healthcare accessibility, workforce availability, and environmental or social stressors to further improve understanding of geographic disparities in mental health outcomes. Extending geographically weighted approaches to alternative spatial scales and longer temporal periods may also provide additional insight into the evolving spatial dynamics of mental health across rural and underserved populations.

## Data Availability

Publicly available datasets were analyzed in this study. This data can be found here: county health rankings & roadmaps. https://www.countyhealthrankings.org/.
