# Supplementary material for: Spatial temporal trends and clustering of mental health in the Great Plains and Rocky Mountains
Source: Front Public Health. 2026 Jul 28;14:1776208. doi: 10.3389/fpubh.2026.1776208 (PMC13457217; doi:10.3389/fpubh.2026.1776208)
Supplement: Supplementary file 1 [file Supplementary_File_1.pdf]

[Supplementary Material]*Supplementary Material*

SUPPLEMENTARY FIGURES AND TABLES

1 GTWR: DEMOGRAPHIC AND HEALTH INDICATORS

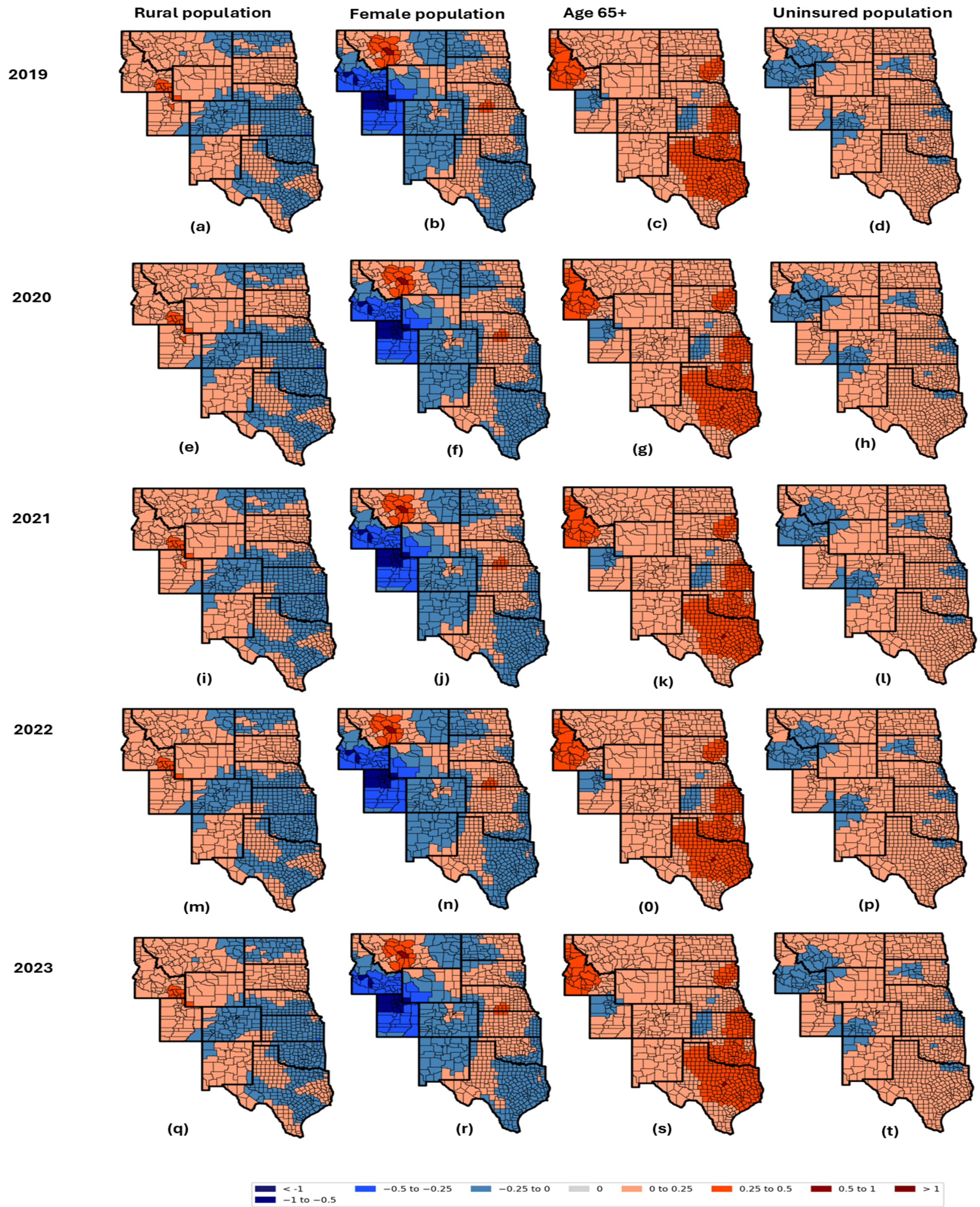

Figure A1. Spatial variation in GTWR coefficients for demographic and health indicators from 2019 to 2023. Panels (a, e, i, m, q) show Rural Population; (b, f, j, n, r) show Female Population; (c, g, k, o, s) show Over Age 65; (d, h, l, p, t) show Uninsured. Red indicates positive associations with frequent mental distress; blue indicates negative associations, with darker tones reflecting stronger coefficient values.

2 GTWR: EDUCATION AND SOCIO-ECONOMIC INDICATORS

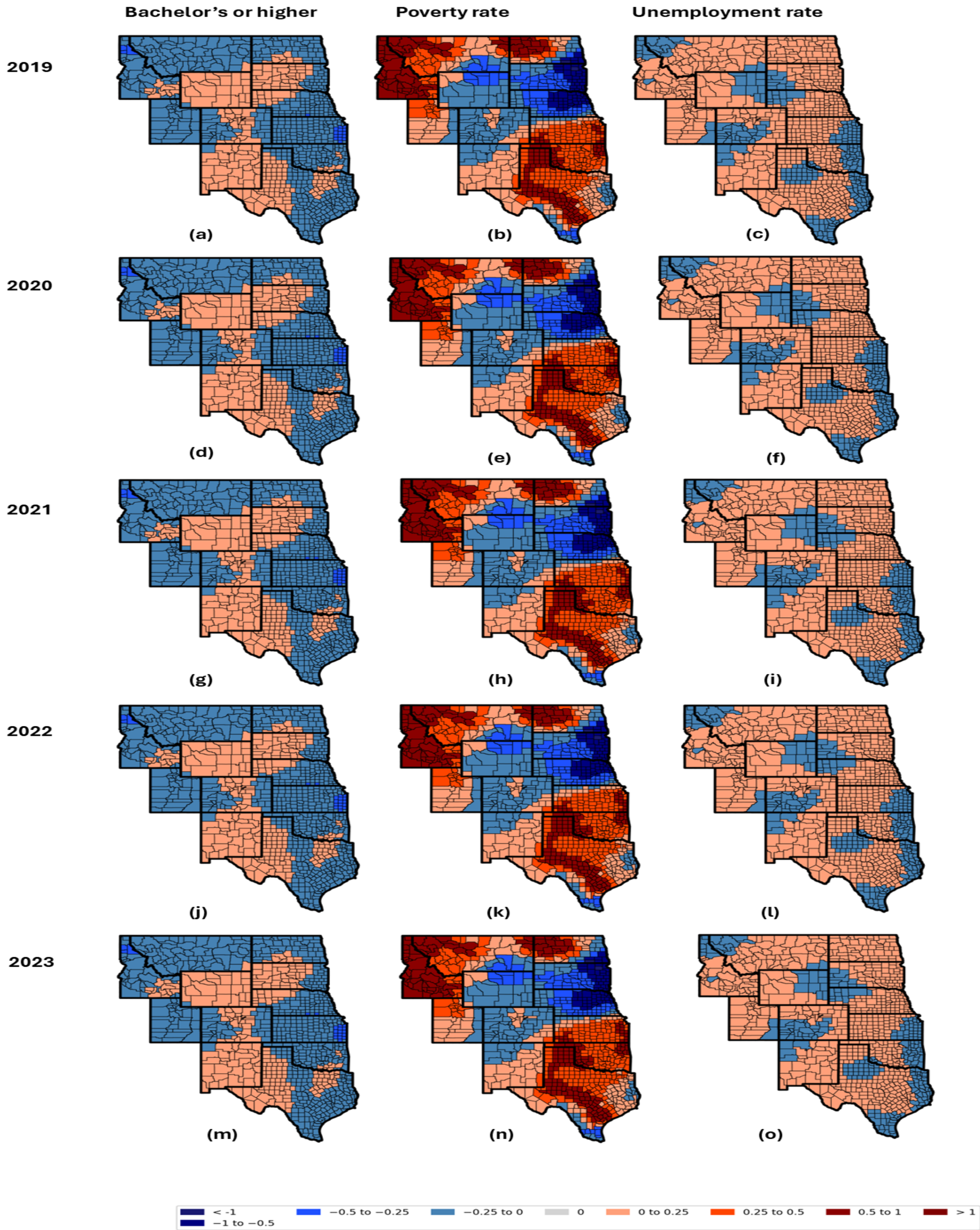

Figure B1. Spatial variation in GTWR coefficients for education and socio-economic indicators from 2019 to 2023. Panels (a, d, g, j, m) show Bachelor's degree or higher; (b, e, h, k, n) show poverty rate; and (c, f, i, l, o) show unemployment rate. Red indicates positive associations and blue indicates negative associations with frequent mental distress.

3 GTWR: RACIAL AND ETHNIC GROUP INDICATORS

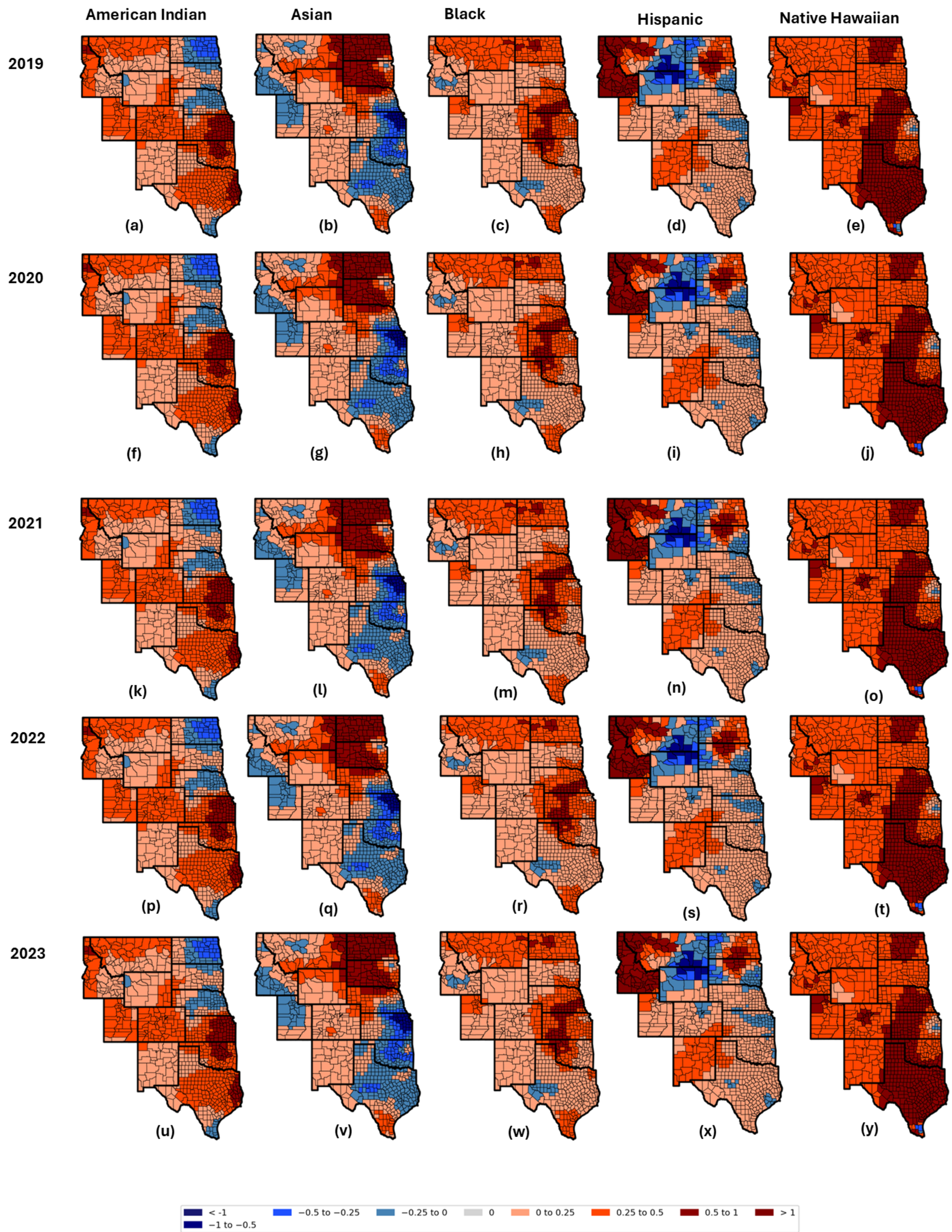

Figure C1. Spatial variation in MGTWR coefficients for racial and ethnic composition variables from 2019 to 2023. Panels (a–e) show American Indian, Asian, Black, Hispanic, and Native Hawaiian populations in 2019; (f–j) show the same variables in 2020; (k–o) in 2021; (p–t) in 2022; and (u–y) in 2023. Red shading indicates positive associations with frequent mental distress, whereas blue shading indicates negative associations, with darker tones reflecting stronger coefficient values. The non-Hispanic White population was used as the reference category.

OLS Diagnostics

To evaluate the adequacy of the global OLS specification, diagnostic tests were performed on the model residuals. The Breusch–Pagan test indicated statistically significant heteroscedasticity in the residuals ($p < 0.001$), suggesting that the variance of the model errors was not constant across counties. In addition, the Durbin–Watson statistic ($DW = 0.590$) indicated substantial positive autocorrelation among residuals. Together, these findings suggest that the assumptions underlying the global OLS model were violated and that important spatial structure remained unexplained. The presence of heteroscedasticity and residual spatial dependence therefore provided additional justification for the use of geographically weighted regression approaches capable of accounting for spatial non-stationarity and localized variation in the associations between frequent mental distress and county-level characteristics.

Local Multicollinearity

To assess localized multicollinearity within the geographically weighted regression framework, local condition numbers (CNs) were calculated following the diagnostic approaches proposed by Wheeler and Tiefelsdorf [10, 25].

The condition numbers exceeding 30 may indicated potentially problematic levels of local multicollinearity [10, 61]. The local multicollinearity diagnostics indicated moderate spatial variability in condition numbers across counties in the Great Plains and Rocky Mountains. The mean local condition number was 22.08, with values ranging from 5.41 to 62.79, as shown in Table 8. A few county-year observations had local condition numbers greater than 30, indicating local multicollinearity in some regions. However, extremely severe instability was not widespread across the study area.

Statistic	Value
Optimal Bandwidth	442.0
Mean Condition Number	22.08
Standard Deviation	12.76
Minimum Condition Number	5.41
25th Percentile	11.00
Median Condition Number	18.00
75th Percentile	32.27
Maximum Condition Number	62.79
County-Year Observations with CN > 30	1,390

Table 8. Summary statistics of local condition numbers used to assess localized multicollinearity within the geographically weighted regression models.

Overall, these findings suggest that localized multicollinearity was present in some counties, particularly in regions where demographic and socioeconomic variables exhibited stronger spatial dependency. Nevertheless, the condition numbers generally remained within ranges commonly reported in geographically weighted regression studies involving population composition variables and regional socioeconomic indicators, suggesting that local coefficient estimates remained reasonably stable across most of the study region.

4 SUMMARY STATISTICS OF LOCAL GTWR COEFFICIENTS BY YEAR

Year	Variable	Mean	SD	Min	Median	Max
**2019**						
2019	Intercept	0.1714	0.4223	-1.0262	0.2267	1.2596
2019	Poverty	0.0271	0.0691	-0.2123	0.0347	0.2109
2019	Unemployment	0.2125	0.1160	-0.1942	0.2108	0.5030
2019	65+ Years	0.2677	0.1570	-0.0649	0.2385	0.6634
2019	Black	0.1622	0.3286	-0.6268	0.0733	2.1714
2019	Hispanic	0.1504	0.4820	-0.6508	0.0424	2.6843
2019	Asian	-0.0068	0.1507	-0.6273	-0.0039	0.5331
2019	Female	-0.0021	0.0820	-0.2756	-0.0041	0.3647
2019	Rural	-0.0542	0.1022	-0.4119	-0.0519	0.2344
2019	Bachelor's+	0.0587	0.0561	-0.1128	0.0592	0.2260
2019	Uninsured	0.2571	0.2301	-0.3069	0.2737	1.1162
2019	American Indian	0.7834	0.7177	-0.4350	0.5091	4.5486
2019	Native Hawaiian	0.0995	0.0988	-0.1745	0.0943	0.3810
**2020**						
2020	Intercept	0.1744	0.4236	-1.0282	0.2299	1.2652
2020	Poverty	0.0270	0.0692	-0.2157	0.0343	0.2126
2020	Unemployment	0.2121	0.1162	-0.1987	0.2107	0.5027
2020	65+ Years	0.2677	0.1572	-0.0653	0.2384	0.6673
2020	Black	0.1622	0.3293	-0.6273	0.0728	2.1781
2020	Hispanic	0.1497	0.4822	-0.6545	0.0415	2.6793
2020	Asian	-0.0069	0.1509	-0.6276	-0.0041	0.5317
2020	Female	-0.0023	0.0822	-0.2818	-0.0041	0.3646
2020	Rural	-0.0541	0.1025	-0.4121	-0.0518	0.2344
2020	Bachelor's+	0.0585	0.0562	-0.1137	0.0586	0.2260
2020	Uninsured	0.2572	0.2306	-0.3067	0.2734	1.1272
2020	American Indian	0.7847	0.7212	-0.4510	0.5083	4.5830
2020	Native Hawaiian	0.0996	0.0990	-0.1758	0.0942	0.3804
**2021**						
2021	Intercept	0.1770	0.4244	-1.0277	0.2333	1.2661
2021	Poverty	0.0269	0.0691	-0.2154	0.0339	0.2127
2021	Unemployment	0.2118	0.1161	-0.1989	0.2100	0.5020
2021	65+ Years	0.2674	0.1571	-0.0655	0.2375	0.6669
2021	Black	0.1622	0.3297	-0.6278	0.0728	2.1797
2021	Hispanic	0.1489	0.4821	-0.6605	0.0394	2.6743
2021	Asian	-0.0071	0.1509	-0.6278	-0.0043	0.5303
2021	Female	-0.0023	0.0823	-0.2835	-0.0041	0.3645
2021	Rural	-0.0539	0.1025	-0.4124	-0.0516	0.2344
2021	Bachelor's+	0.0584	0.0561	-0.1135	0.0583	0.2260
2021	Uninsured	0.2572	0.2308	-0.3066	0.2733	1.1307
2021	American Indian	0.7849	0.7227	-0.4670	0.5072	4.5998
2021	Native Hawaiian	0.0996	0.0990	-0.1764	0.0942	0.3797
**2022**						
2022	Intercept	0.1794	0.4250	-1.0273	0.2372	1.2650
2022	Poverty	0.0268	0.0689	-0.2134	0.0336	0.2127
2022	Unemployment	0.2115	0.1159	-0.1967	0.2093	0.5013
2022	65+ Years	0.2671	0.1569	-0.0656	0.2372	0.6665
2022	Black	0.1623	0.3300	-0.6283	0.0719	2.1813
2022	Hispanic	0.1480	0.4818	-0.6665	0.0385	2.6693
2022	Asian	-0.0074	0.1508	-0.6280	-0.0044	0.5288
2022	Female	-0.0023	0.0822	-0.2822	-0.0042	0.3643

Continued on next page

Year	Variable	Mean	SD	Min	Median	Max
2022	Rural	-0.0536	0.1024	-0.4094	-0.0514	0.2344
2022	Bachelor's+	0.0582	0.0560	-0.1133	0.0580	0.2260
2022	Uninsured	0.2571	0.2309	-0.3064	0.2728	1.1305
2022	American Indian	0.7845	0.7231	-0.4830	0.5065	4.5946
2022	Native Hawaiian	0.0995	0.0990	-0.1763	0.0942	0.3791
**2023**						
2023	Intercept	0.1814	0.4250	-1.0244	0.2415	1.2640
2023	Poverty	0.0267	0.0686	-0.2078	0.0341	0.2110
2023	Unemployment	0.2113	0.1155	-0.1914	0.2090	0.5001
2023	65+ Years	0.2665	0.1564	-0.0654	0.2377	0.6617
2023	Black	0.1623	0.3300	-0.6288	0.0721	2.1777
2023	Hispanic	0.1471	0.4812	-0.6742	0.0372	2.6642
2023	Asian	-0.0078	0.1506	-0.6283	-0.0045	0.5274
2023	Female	-0.0022	0.0819	-0.2762	-0.0043	0.3642
2023	Rural	-0.0533	0.1021	-0.4065	-0.0508	0.2344
2023	Bachelor's+	0.0580	0.0557	-0.1120	0.0577	0.2261
2023	Uninsured	0.2568	0.2306	-0.3062	0.2730	1.1227
2023	American Indian	0.7830	0.7215	-0.4991	0.5068	4.5713
2023	Native Hawaiian	0.0993	0.0988	-0.1754	0.0944	0.3784

Table 9 Summary statistics of local GTWR coefficients by year. Values represent the distribution of local coefficients across counties in the Great Plains and Rocky Mountain regions.

5 SUMMARY STATISTICS OF LOCAL MGTWR COEFFICIENTS BY YEAR

Year	Variable	Mean	SD	Min	Median	Max
**2019**						
2019	Intercept	0.1886	0.4136	-0.9473	0.2759	1.0825
2019	Poverty	0.0196	0.0740	-0.2406	0.0320	0.2126
2019	Unemployment	0.2125	0.0960	-0.0665	0.2082	0.4407
2019	65+ Years	0.2790	0.1310	-0.0336	0.2547	0.5664
2019	Black	0.1644	0.2525	-0.2134	0.0789	1.3667
2019	Hispanic	0.1573	0.4249	-0.6119	0.0657	1.9500
2019	Asian	0.0067	0.1091	-0.5167	0.0222	0.2451
2019	Female	0.0010	0.0450	-0.1134	0.0059	0.1052
2019	Rural	-0.0509	0.0733	-0.2125	-0.0433	0.1177
2019	Bachelor's+	0.0694	0.0584	-0.1065	0.0674	0.2496
2019	Uninsured	0.2546	0.2121	-0.3268	0.2483	0.8965
2019	Native Hawaiian	0.0986	0.0866	-0.1211	0.1014	0.3606
2019	American Indian	0.8354	0.6435	0.1580	0.5316	3.5053
**2020**						
2020	Intercept	0.1893	0.4138	-0.9483	0.2760	1.0835
2020	Poverty	0.0196	0.0739	-0.2407	0.0320	0.2125
2020	Unemployment	0.2125	0.0961	-0.0668	0.2081	0.4407
2020	65+ Years	0.2790	0.1311	-0.0338	0.2548	0.5665
2020	Black	0.1643	0.2526	-0.2139	0.0786	1.3667
2020	Hispanic	0.1571	0.4250	-0.6134	0.0660	1.9503
2020	Asian	0.0067	0.1091	-0.5168	0.0222	0.2451
2020	Female	0.0010	0.0450	-0.1133	0.0059	0.1052
2020	Rural	-0.0509	0.0733	-0.2125	-0.0434	0.1177
2020	Bachelor's+	0.0694	0.0584	-0.1065	0.0674	0.2495
2020	Uninsured	0.2546	0.2122	-0.3273	0.2483	0.8965

Continued on next page

Year	Variable	Mean	SD	Min	Median	Max
2020	Native Hawaiian	0.0985	0.0866	-0.1213	0.1014	0.3605
2020	American Indian	0.8348	0.6433	0.1532	0.5308	3.5063
**2021**						
2021	Intercept	0.1900	0.4140	-0.9489	0.2761	1.0844
2021	Poverty	0.0196	0.0739	-0.2409	0.0320	0.2124
2021	Unemployment	0.2124	0.0962	-0.0671	0.2080	0.4407
2021	65+ Years	0.2790	0.1311	-0.0340	0.2549	0.5664
2021	Black	0.1642	0.2526	-0.2143	0.0784	1.3667
2021	Hispanic	0.1569	0.4251	-0.6146	0.0660	1.9501
2021	Asian	0.0067	0.1090	-0.5168	0.0222	0.2449
2021	Female	0.0010	0.0450	-0.1132	0.0059	0.1052
2021	Rural	-0.0509	0.0733	-0.2126	-0.0434	0.1177
2021	Bachelor's+	0.0693	0.0584	-0.1065	0.0674	0.2493
2021	Uninsured	0.2546	0.2123	-0.3277	0.2482	0.8964
2021	Native Hawaiian	0.0985	0.0866	-0.1214	0.1014	0.3603
2021	American Indian	0.8342	0.6431	0.1488	0.5309	3.5066
**2022**						
2022	Intercept	0.1907	0.4139	-0.9471	0.2764	1.0847
2022	Poverty	0.0195	0.0739	-0.2410	0.0320	0.2124
2022	Unemployment	0.2123	0.0962	-0.0674	0.2079	0.4407
2022	65+ Years	0.2790	0.1311	-0.0341	0.2549	0.5664
2022	Black	0.1640	0.2526	-0.2143	0.0783	1.3663
2022	Hispanic	0.1566	0.4252	-0.6158	0.0654	1.9498
2022	Asian	0.0066	0.1090	-0.5168	0.0222	0.2448
2022	Female	0.0010	0.0450	-0.1131	0.0059	0.1052
2022	Rural	-0.0508	0.0733	-0.2126	-0.0433	0.1177
2022	Bachelor's+	0.0693	0.0584	-0.1065	0.0674	0.2492
2022	Uninsured	0.2545	0.2123	-0.3277	0.2481	0.8957
2022	Native Hawaiian	0.0984	0.0866	-0.1216	0.1014	0.3602
2022	American Indian	0.8333	0.6425	0.1470	0.5309	3.5028
**2023**						
2023	Intercept	0.1913	0.4138	-0.9449	0.2768	1.0848
2023	Poverty	0.0195	0.0738	-0.2411	0.0320	0.2123
2023	Unemployment	0.2123	0.0963	-0.0677	0.2079	0.4408
2023	65+ Years	0.2790	0.1311	-0.0342	0.2549	0.5662
2023	Black	0.1639	0.2525	-0.2142	0.0781	1.3658
2023	Hispanic	0.1564	0.4251	-0.6165	0.0649	1.9490
2023	Asian	0.0066	0.1090	-0.5167	0.0222	0.2446
2023	Female	0.0010	0.0450	-0.1131	0.0059	0.1051
2023	Rural	-0.0508	0.0733	-0.2126	-0.0433	0.1177
2023	Bachelor's+	0.0692	0.0583	-0.1064	0.0674	0.2491
2023	Uninsured	0.2544	0.2122	-0.3277	0.2481	0.8949
2023	Native Hawaiian	0.0984	0.0866	-0.1218	0.1014	0.3600
2023	American Indian	0.8325	0.6419	0.1458	0.5306	3.4983

Table 10 Summary statistics of local MGTWR coefficients by year. Values represent the distribution of local coefficients across counties in the Great Plains and Rocky Mountain regions.
